# Use of ionizing radiation in a Norwegian cohort of children with congenital heart disease: imaging frequency and radiation dose for the Health Effects of Cardiac Fluoroscopy and Modern Radiotherapy in Pediatrics (HARMONIC) study

**DOI:** 10.1007/s00247-023-05774-8

**Published:** 2023-09-29

**Authors:** Susmita Afroz, Bjørn H. Østerås, Utheya S. Thevathas, Gaute Dohlen, Caroline Stokke, Trude E. Robsahm, Hilde M. Olerud

**Affiliations:** 1 Department of Optometry, Radiography and Lighting Design, University of South-Eastern Norway, Grønland 58, Drammen, Norway; 2 Department of Pediatric Cardiology, Oslo University Hospital, Oslo, Norway; 3 Department of Physics and Computational Radiology, Oslo University Hospital, Oslo, Norway; 4 Department of Physics, University of Oslo, Oslo, Norway; 5 Research Department, Cancer Registry of Norway, Oslo, Norway

**Keywords:** Congenital, Heart defects, Pediatric, Radiation dose, Radiology

## Abstract

**Background:**

The European-funded Health Effects of Cardiac Fluoroscopy and Modern Radiotherapy in Pediatrics (HARMONIC) project is a multicenter cohort study assessing the long-term effects of ionizing radiation in patients with congenital heart disease. Knowledge is lacking regarding the use of ionizing radiation from sources other than cardiac catheterization in this cohort.

**Objective:**

This study aims to assess imaging frequency and radiation dose (excluding cardiac catheterization) to patients from a single center participating in the Norwegian HARMONIC project.

**Materials and methods:**

Between 2000 and 2020, we recruited 3,609 patients treated for congenital heart disease (age < 18 years), with 33,768 examinations categorized by modality and body region. Data were retrieved from the radiology information system. Effective doses were estimated using International Commission on Radiological Protection Publication 60 conversion factors, and the analysis was stratified into six age categories: newborn; 1 year, 5 years, 10 years, 15 years, and late adolescence.

**Results:**

The examination distribution was as follows: 91.0% conventional radiography, 4.0% computed tomography (CT), 3.6% diagnostic fluoroscopy, 1.2% nuclear medicine, and 0.3% noncardiac intervention. In the newborn to 15 years age categories, 4–12% had ≥ ten conventional radiography studies, 1–8% underwent CT, and 0.3–2.5% received nuclear medicine examinations. The median effective dose ranged from 0.008–0.02 mSv and from 0.76–3.47 mSv for thoracic conventional radiography and thoracic CT, respectively. The total effective dose burden from thoracic conventional radiography ranged between 28–65% of the dose burden from thoracic CT in various age categories (40% for all ages combined). The median effective dose for nuclear medicine lung perfusion was 0.6–0.86 mSv and for gastrointestinal fluoroscopy 0.17–0.27 mSv. Because of their low frequency, these procedures contributed less to the total effective dose than thoracic radiography.

**Conclusion:**

This study shows that CT made the largest contribution to the radiation dose from imaging (excluding cardiac intervention). However, although the dose per conventional radiograph was low, the large number of examinations resulted in a substantial total effective dose. Therefore, it is important to consider the frequency of conventional radiography while calculating cumulative dose for individuals. The findings of this study will help the HARMONIC project to improve risk assessment by minimizing the uncertainty associated with cumulative dose calculations.

**Graphical Abstract:**

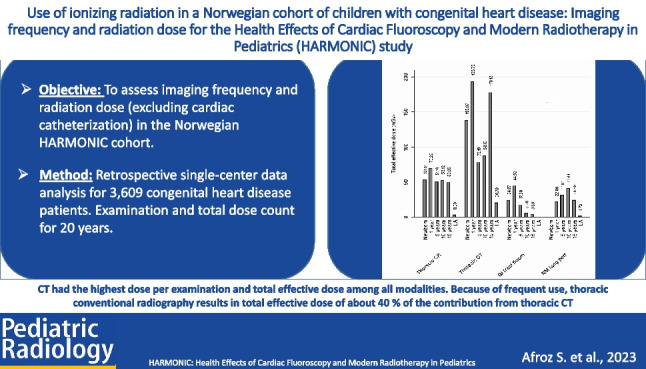

**Supplementary Information:**

Supplementary material is available at 10.1007/s00247-023-05774-8.

## Introduction

Congenital heart disease (CHD) is the most prevalent type of major birth defect, occurring in approximately 0.8% of live births worldwide [1]. Many individuals with CHD undergo life-saving cardiac catheterization procedures that result in substantial exposure to ionizing radiation, with frequent cumulative organ doses greater than 1,000 mGy [2, 3]. The radiation dose to the lungs and heart generally ranges from 5–20 mGy, occasionally exceeding 100 mGy [4–7]. In addition, many reports raise concern that the frequency of computed tomography (CT) examinations for patients with CHD is increasing [8, 9]. This could lead to an increased radiation dose, even though the dose per CT examination is decreasing, especially in the cardiac region due to faster scans and the possibility of imaging the entire heart in a single heartbeat [10, 11]. With the increasing long-term survival of these patients, there is growing concern about the potential late health effects of radiation exposure [12], particularly since children are more susceptible to radiation effects and damage than adults [12, 13].

The estimation of risks associated with ionizing radiation is largely based on models derived from atomic bomb survivors, nuclear workers, groups of individuals treated using radiotherapy, or occupational exposure [14, 15]. In recent years, a few pediatric cohorts have been used to study the long-term health effects of radiation exposure from pediatric CT and cardiac fluoroscopy, indicating increased cancer risk and mortality [16–20].

As children with CHD are exposed to substantial amounts of ionizing radiation, they are a suitable group for studies on the later health effects of radiation. Recently, these patients have become the focus of the European Union-funded multicenter Health Effects of Cardiac Fluoroscopy and Modern Radiotherapy in Pediatrics (HARMONIC) study (https://harmonicproject.eu/) (2019–2024). The HARMONIC project’s objective is to develop a European cohort of children with CHD for long-term follow-up. The project aims to provide estimates of radiation exposure and associated risks of radiation-induced health effects [14]. The dosimetric approach involves thorough individual assessments of radiation doses from cardiac catheterization procedures. However, these children are exposed to other sources of medical ionizing radiation, both related and unrelated to their CHD. These additional exposures need to be considered in the epidemiological analysis of late effects of radiation; otherwise, patients’ risk from the procedures related to CHD may be overestimated. Published data on examination frequency and doses are limited [13, 21–23]. Therefore, both age-specific and cohort-specific information regarding additional examinations is required. Such an overview would also be beneficial for clinicians and hospitals when evaluating the cost and benefit of patient imaging follow-up and to increase awareness of the cumulative radiation dose and dose contribution from different modalities.

The objective of this study was to determine the frequency and radiation dose contribution of conventional radiography, CT, diagnostic fluoroscopy, nuclear medicine (NM), and noncardiac intervention among Norwegian patients with CHD, stratified by age. The major contributors by modality and examination region were compared to identify the major sources of radiation exposure. These data will provide input to the multicenter HARMONIC project and facilitate further risk assessment related to radiation exposure.

## Materials and methods

### Norwegian cohort and data source

This retrospective study was approved by the Norwegian regional committees for medical and health research ethics (REK) and institutional data protection officer (PVO). As part of the HARMONIC project, we are establishing a Norwegian national cohort of patients with CHD based on the criteria set by HARMONIC [14]. This includes patients who have undergone at least one cardiac catheterization procedure before the age of 18 years, which may be interventional or diagnostic. Patient records were obtained from the local patient registers at the Department of Pediatric Cardiology, Oslo University Hospital. The cohort represents national data, as Oslo University Hospital plays a national role in pediatric cardiology. From 1990 to 2020, the cohort included 4,086 patients.

**Fig. 1:**
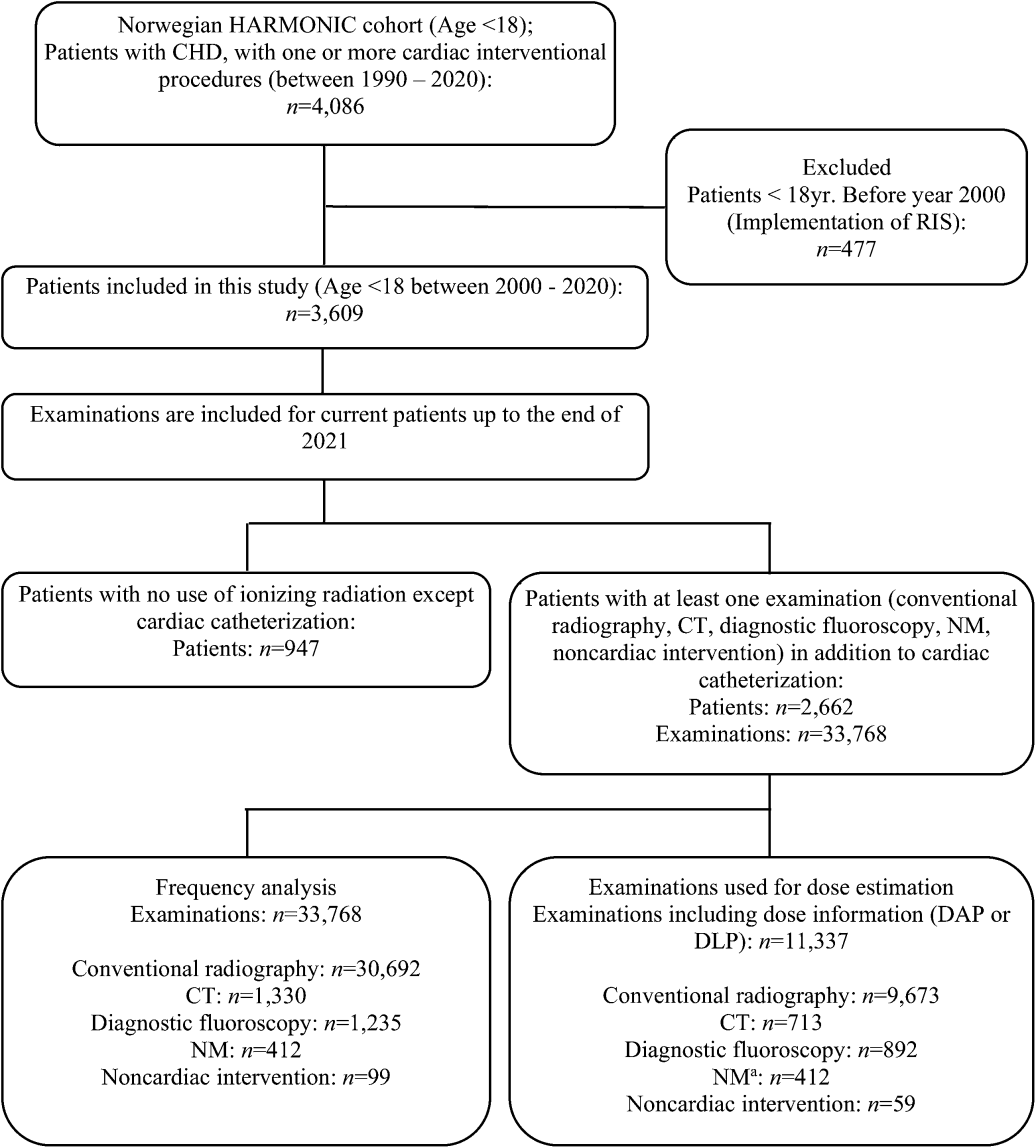
Flowchart showing the study inclusion criteria. ^a^Dose estimations for nuclear medicine were performed for all procedures using population-weighted administered activity following the European Association of Nuclear Medicine dosage card version 5.7.2016 method. *CHD* congenital heart disease, *CT* computed tomography, *DAP* dose area product, *DLP* dose length product, *HARMONIC* Health Effects of Cardiac Fluoroscopy and Modern Radiotherapy in Pediatrics, *NM* nuclear medicine, *RIS* radiology information system

The flowchart (Fig. 1) shows the inclusion of patients and examinations in this study. Of the current Norwegian HARMONIC cohort of 4,086 patients, 3,609 were under the age of 18 years between 2000 and 2021. In this study, examinations were included up to the end of 2021, but the inclusion of new patients was stopped in 2020. This cut-off point was chosen as it covers all examinations recorded in the radiology information system (RIS) from 2000 to 2021.

We retrieved data on 33,768 examinations (conventional radiography, CT, diagnostic fluoroscopy, NM, and noncardiac intervention) from the RIS, including age at examination, sex, examination name and date, dose area product (DAP), dose length product (DLP), and fluoroscopy time. The available records of the machine models and manufacturers are provided in Supplementary Material 1. A total of 9,673 (out of 30,692) conventional radiography, 892 (out of 1,235) diagnostic fluoroscopy, and 59 (out of 100) noncardiac interventional examinations reporting DAP, 713 (out of 1,330) CT examinations reporting DLP, and all 412 NM examinations using population-weighted administered activity were used for dose calculation. There is no obvious time pattern for the missing data.

### Categorization of radiological procedures

The examination frequency and probability were counted for each modality: conventional radiography, CT, diagnostic fluoroscopy, NM, and noncardiac intervention. Examination type and body region were classified according to the European Commission DOSE DATAMED methodology (RP154, RP180), except for NM, as radioactivity is injected [24, 25].

### Comparison of doses by modality

To compare doses from different modalities, we used published conversion factors to estimate the effective dose for pediatric patients, all based on the International Commission on Radiological Protection (ICRP) Publication 60 recommendation [26]. For conventional radiography, diagnostic fluoroscopy, and noncardiac intervention, the conversion factors were based on the DAP [27], whereas for CT, they were based on DLP [28]. For NM, we used the weight chart by age for Norwegian children from the study conducted by Júlíusson et al. [29] and calculated the administered activity following the European Association of Nuclear Medicine (EANM) dosage card version 5.7.2016 [30]. Conversion factors between the administered activity and effective dose for NM examinations were obtained from ICRP Publication 128 [31]. To assess the total radiation exposure for this cohort, we estimated the total effective dose given to all patients in each of the age categories over the study period (2000–2020), based on the median effective dose per examination and the respective examination frequency of various types and modalities. It should be noted that, within HARMONIC, the estimation of organ doses from cardiac catheterization procedures will be performed as described by Harbron et al. [14]. Therefore, they were not included in this study.

### Data analysis

The analyses were performed across six age categories according to the HARMONIC study protocol—newborn (0–3 months), 1 year (4–30 months), 5 years (31–90 months), 10 years (91–150 months), 15 years (151–210 months), and late adolescence (211–216 months)—based on the patient’s age at the time of examination. Data analyses were conducted using Stata version 17 (StataCorp LLC, College Station, TX) [32]. Descriptive statistics were used to summarize the radiation exposure data. The probability of a patient undergoing an examination using a given modality was calculated across different age categories. The probability is presented here as the proportion of the total number of patients.

## Results

### Frequency and probability by modality

The number of patients and examinations stratified by modality and age is shown in Table 1. Of the 3,609 patients, 2,662 (73.8%) were imaged using one or more of the following procedures: conventional radiography, CT, diagnostic fluoroscopy, NM, or noncardiac intervention. The remaining patients were examined using ultrasound, magnetic resonance imaging, or not at all. The most used modality was conventional radiography (about 91% of examinations), while CT (about 3.9%) and diagnostic fluoroscopy (3.4%) were also used. NM (1.2%) and noncardiac intervention (0.3%) were used less frequently. The examination frequency by modality varied across age categories, with conventional radiography and diagnostic fluoroscopy being used more frequently for young patients and decreasing with age, whereas CT, NM, and noncardiac intervention showed no clear trend.

**Table 1:** Examination frequency stratified by age and modality for the Health Effects of Cardiac Fluoroscopy and Modern Radiotherapy in Pediatrics (HARMONIC) cohort of Norwegian patients with congenital heart disease

Age category^a^	Number of patients examined^b^ (%)	Total number of examinations				
		Conventional radiography	CT	Diagnostic fluoroscopy	NM	Noncardiac intervention
0–3 m (newborn) (*n* =1,773)	561 (31.6%)	4,785	178	157	20	16
4–30 m (1 year) (*n* =2,111)	1,240 (58.7%)	9,539	293	410	86	29
31–90 m (5 years) (*n* =2,782)	1,195 (43%)	6,894	248	323	103	22
91–150 m (10 years) (*n* =3,100)	830 (26.8%)	4,715	292	204	119	7
151–210 m (15 years) (*n* =2,954)	884 (29.9%)	4,492	297	137	74	24
211–216 m (late adolescents) (*n* =2,473)	163 (6.6%)	267	22	4	10	1
	Total	30,692	1,330	1,235	412	99

^a^Some patients may fall into multiple age categories if procedures were performed at different ages. ^b^Patients who had at least one conventional radiography, CT, diagnostic fluoroscopy, NM, or noncardiac intervention

*CT* computed tomography, *m* months, *NM* nuclear medicine

Many patients underwent multiple examinations using the same modality within the same age category (Table 2). A substantial proportion of patients (20–45% across age categories up to 210 months) received 1–9 conventional radiography examinations. Over 10% of children in both the newborn and the 4–30 months category had ten or more such examinations; this frequency was reduced for older patients. For CT, 5–7.5% of patients had at least one CT, with 1.8–3% having multiple in each age category (up to 210 months). For diagnostic fluoroscopy, approximately 3–9% of patients had an examination in each age category, with most occurring at 4–30 months (about 4% of patients had multiple procedures) and 31–90 months (about 2% had multiple procedures). NM examinations were approximately 1–2.5% of patients (in each age category up to 210 months). Noncardiac intervention was performed on less than 1% of patients in each age category.

**Table 2:** Probability distribution of examinations by modality in six age categories for the Health Effects of Cardiac Fluoroscopy and Modern Radiotherapy in Pediatrics (HARMONIC) cohort of Norwegian patients with congenital heart disease

Age category	Number of examinations	Proportion of patients				
		Conventional radiography	CT	Diagnostic fluoroscopy	NM	Noncardiac intervention
0–3 m (newborn) (*n* =1,773)	0	69.1	92.4	96.0	99.0	99.2
	1	5.4	5.8	1.8	0.9	0.8
	2–9	15.4	1.8	2.1	0.1	0.1
	≥ 10	10.1	0.0	0.1	0.0	0.0
4–30 m (1 year) (*n* =2,111)	0	41.9	92.3	91.0	97.5	99.1
	1	7.9	4.6	4.8	1.4	0.6
	2–9	38.0	3.1	4.1	1.1	0.3
	≥ 10	12.2	0.0	0.1	0.0	0.0
31–90 m (5 years) (*n* =2,782)	0	58.1	94.6	94.0	97.6	99.4
	1	8.2	3.7	3.9	1.6	0.5
	2–9	27.6	1.7	1.9	0.8	0.1
	≥ 10	6.1	0.0	0.1	0.0	0.0
91–150 m (10 years) (*n* =3,100)	0	74.1	94.9	96.7	97.6	99.8
	1	6.7	3.0	1.9	1.5	0.2
	2–9	15.1	2.0	1.3	0.8	0.0
	≥ 10	4.1	0.0	0.1	0.0	0.0
151–210 m (15 years) (*n* =2,954)	0	71.3	93.6	97.1	98.1	99.5
	1	7.4	4.6	2.0	1.4	0.3
	2–9	17.4	1.8	0.9	0.5	0.2
	≥ 10	4.0	0.0	0.0	0.0	0.0
211–216 m (late adolescents) (*n* =2,473)	0	93.9	99.2	99.8	99.7	99.96
	1	4.1	0.7	0.2	0.2	0.04
	2–9	1.9	0.1	0.0	0.1	0.0
	≥ 10	0.0	0.0	0.0	0.0	0.0

*CT* computed tomography, *m* months, *NM* nuclear medicine

### Frequent examination types

The most frequent examinations for conventional radiography, CT, diagnostic fluoroscopy, and NM are shown in Table 3. Approximately 90.1% of the 30,692 conventional radiography examinations (on 2,639 [73.1%] patients) were anteroposterior and lateral thorax (52%), anteroposterior thorax (45%), spine for scoliosis (2%), and abdominal radiographs (1%). Nearly 52.5% of the 1,330 CT examinations (on 580 [16.1%] patients) were performed on the thorax (58%), head (35%), and abdomen (7%). A patient receiving a head CT usually had more than two examinations, while those that had a thoracic CT received an average of 1.7 examinations. Of the 1,235 diagnostic fluoroscopy examinations (on 485 [13.4%] patients), 31.1% were performed on the chest/thorax and esophagus. Of the 412 NM examinations (on 214 [5.9%] patients), 58.3% were lung perfusion and myocardial perfusion studies.

**Table 3:** Most frequent examinations for each modality in the Health Effects of Cardiac Fluoroscopy and Modern Radiotherapy in Pediatrics (HARMONIC) cohort of Norwegian patients with congenital heart disease

Examination modality	Most frequent examinations	Number of examinations	Number of patients
Conventional radiography	AP and lateral thorax	14,421	2,393
	AP thorax	12,416	958
	Scoliosis	507	102
	Abdomen	299	172
CT	Thorax	403	234
	Head	244	117
	Abdomen	51	40
Diagnostic fluoroscopy	Chest/thorax	214	152
	Esophagus	170	92
NM	Lung perfusion	181	105
	Myocardial perfusion	59	38
Noncardiac intervention	NA	-	-

*AP *anteroposterior,* CT* computed tomography, *NA* not available, *NM* nuclear medicine

The probability distributions of the four most common radiological examinations (radiographs of the thorax, CT thorax, fluoroscopy thorax, and NM lung perfusion) by age category are presented in Table 4. Notably, patients in the 4‒30 months and 31‒90 months categories had a higher likelihood of having three or more thoracic radiographs compared to those in the other age categories.

**Table 4:** Probability distribution of the four most common examination types from different modalities in six age categories for the Health Effects of Cardiac Fluoroscopy and Modern Radiotherapy in Pediatrics (HARMONIC) cohort of Norwegian patients with congenital heart disease

Age category	Number of examinations	Proportion of patients			
		X-ray thorax	CT thorax	Fluoroscopy thorax	NM lung perfusion
0–3 m (newborn) (*n* =1,773)	0	82.5	97.9	99.8	99.8
	1	8.4	1.9	0.2	0.2
	2	4.8	0.2	0.1	0.1
	≥ 3–11	4.3	0.1	0.0	0.0
4–30 m (1 year) (*n* =2,111)	0	49.9	96.1	97.8	98.7
	1	7.6	2.6	1.7	1.0
	2	13.8	0.6	0.4	0.2
	≥ 3–26	28.7	0.7	0.1	0.0
31–90 m (5 years) (*n* =2,782)	0	63.5	97.8	97.7	98.6
	1	7.3	1.5	1.8	1.1
	2	9.5	0.4	0.4	0.2
	≥ 3–45	19.7	0.3	0.1	0.1
91–150 m (10 years) (*n* =3,100)	0	78.1	98.5	99.2	98.8
	1	6.2	1.0	0.5	0.8
	2	4.0	0.4	0.2	0.2
	≥ 3–38	11.7	0.2	0.1	0.2
151–210 m (15 years) (*n* =2,954)	0	75.1	98.3	99.5	99.3
	1	7.3	1.4	0.5	0.4
	2	5.4	0.3	0.0	0.2
	≥ 3–23	12.2	0.0	0.0	0.0
211–216 m (late adolescents) (*n* =2,473)	0	95.2	99.8	99.96	99.96
	1	3.4	0.2	0.04	0.0
	2	0.9	0.0	0.0	0.04
	≥ 3–12	0.5	0.0	0.0	0.0

*CT* computed tomography, *m* months, *NM* nuclear medicine

### Effective dose by modality, body region and age category

Most examinations were conducted on the thoracic region. The DAP or DLP and the effective dose per examination for thoracic conventional radiography, thoracic CT, and gastrointestinal fluoroscopy are displayed in Table 5. The effective dose was highest for CT scans across all age categories.

**Table 5:** Median and interquartile range of dose area product, dose length product and effective dose values in conventional radiography, computed tomography and diagnostic fluoroscopy performed mostly of the thoracic region for the Health Effects of Cardiac Fluoroscopy and Modern Radiotherapy in Pediatrics (HARMONIC) cohort of Norwegian patients with congenital heart disease

Modality	Body Region	Age category	Number of examinations	Number of examinations with DAP/DLP	Median (IQR) DAP (Gycm^2^)	Median (IQR) DLP (mGycm)	Median (IQR) effective dose (mSv)
Conventional radiography	Thorax	0–3 m (newborn)	4,615	428	0.01 (0.004–0.01)		0.012 (0.01–0.02)
		4–30 m (1 year)	9,322	2,376	0.01 (0.01–0.02)		0.008 (0.005–0.01)
		31–90 m (5 years)	6,567	2,780	0.02 (0.01–0.04)		0.008 (0.004–0.02)
		91–150 m (10 years)	4,216	1,910	0.05 (0.03–0.07)		0.013 (0.01–0.02)
		151–210 m (15 years)	3,840	1,323	0.08 (0.06–0.12)		0.013 (0.01–0.02)
		211–216 m (late adolescents)	177	19	0.13 (0.1–0.17)		0.020 (0.02–0.03)
CT	Thorax	0–3 m (newborn)	58	27		61.04 (43.1–65.65)	2.38 (1.68–2.56)
		4–30 m (1 year)	138	51		54.00 (23–88.65)	1.40 (0.59–2.30)
		31–90 m (5 years)	104	48		42.30 (25.81–69.73)	0.76 (0.46–1.26)
		91–150 m (10 years)	94	44		72.14 (36.36–126.65)	0.94 (0.47–1.65)
		151–210 m (15 years)	75	41		169.65 (58–248.15)	2.38 (0.81–3.47)
		211–216 m (late adolescents)	6	2		247.50 (154–341)	3.47 (2.16–4.77)
Diagnostic fluoroscopy	Gastrointestinal tract (neck + chest + abdomen)	0–3 m (newborn)	91	74	0.15 (0.07–0.27)		0.27 (0.12–0.48)
		4–30 m (1 year)	197	121	0.30 (0.15–0.50)		0.23 (0.11–0.38)
		31–90 m (5 years)	83	63	0.44 (0.19–0.78)		0.22 (0.09–0.38)
		91–150 m (10 years)	32	25	0.54 (0.18–0.99)		0.18 (0.06–0.33)
		151–210 m (15 years)	23	15	0.77 (0.28–1.24)		0.17 (0.06–0.27)
		211–216 m (late adolescents)	0	0	-		-

*CT* computed tomography, *DAP* dose area product, *DLP* dose length product, *IQR* interquartile range, *m* months

The DAP, DLP, and effective dose per examination for conventional radiography, CT, diagnostic fluoroscopy, and noncardiac intervention stratified by body region (except noncardiac intervention) and age category are available in Supplementary Material 2. Median DAP and effective dose values were consistent across age for conventional radiography, except for the trunk region, where DAP was higher for patients between ten years and late adolescence than for younger patients. The median effective dose from head CT was largest for newborns (2.59 mSv) and decreased to 0.38 mSv for late adolescents. For diagnostic fluoroscopy, the effective dose values did not differ substantially between the gastrointestinal (0.17–0.27 mSv) and urogenital tracts (0.10–0.22 mSv). However, all angiography (excluding the heart) resulted in greater DAP and fluoroscopy time values than other fluoroscopy examinations, across all age categories. Because of the small number of examinations, noncardiac interventions were grouped together, regardless of body region. The DAP values for noncardiac interventions increased with age, likely due to increased weight with age.

The effective dose from NM examinations depends on radiopharmaceutical and age; the latter is also used to estimate injected activity from age-specific population weight. The highest effective dose was estimated at 9–15 mSv for lung ventilation using 99mTc-labeled technegas, whereas the lowest dose was estimated for renography using 99mTc-labeled mercaptoacetyl triglycine (MAG3), assuming normal renal function (Table 6).

**Table 6:** Estimation of dose from most frequent nuclear medicine examinations for the Health Effects of Cardiac Fluoroscopy and Modern Radiotherapy in Pediatrics (HARMONIC) cohort of Norwegian patients with congenital heart disease

Examination	Age category	Radio-pharmaceutical	Weight range (kg)	Administered activity range (MBq)	Conversion factor-effective dose/administered activity (mSv/MBq)^a^	Effective dose range (mSv)
Lung perfusion	0–3 m (newborn)	^99m^Tc-labeled MAA	3.7–6.5	10	NA	-
	4–30 m (1 year)		6.5–12.6	10–17.6	0.063	0.63–1.11
	31–90 m (5 years)		12.6–24.4	17.6–31.9	0.034	0.60–1.09
	91–150 m (10 years)		24.4–42	31.9–51.2	0.023	0.74–1.18
	151–210 m (15 years)		42–69.1	51.2–78.4	0.016	0.82–1.25
	211–216 m (late adolescents)		> 69.1	≥ 78.4	0.011	≥ 0.86
Lung ventilation	0–3 m (newborn)	^99m^Tc-labeled technegas	3.7–6.5	100	NA	-
	4–30 m (1 year)		6.5–12.6	100–153.9	0.087	8.70–13.39
	31–90 m (5 years)		12.6–24.4	153.9–279.8	0.047	7.23–13.15
	91–150 m (10 years)		24.4–42	279.8–447.9	0.031	8.67–13.88
	151–210 m (15 years)		42–69.1	447.9–686	0.022	9.85–15.09
	211–216 m (late adolescents)		> 69.1	≥ 686	0.015	≥ 10.29
Myocardial scintigraphy, Stress	0–3 m (newborn)	^99m^Tc-labeled sestamibi/tetrofosmin2	3.7–6.5	84–143.6	NA	-
	4–30 m (1 year)		6.5–12.6	143.6–263.8	0.045^b^	6.46–11.86
	31–90 m (5 years)		12.6–24.4	263.8–479.6	0.023^b^	6.06–11.03
	91–150 m (10 years)		24.4–42	479.6–767.8	0.016^b^	7.67–12.28
	151–210 m (15 years)		42–69.1	767.8–1176	0.01^b^	7.67–11.76
	211–216 m (late adolescents)		> 69.1	≥ 1176	0.0079^b^	≥ 9.29
Myocardial scintigraphy, Rest	0–3 m (newborn)	^99m^Tc-labeled sestamibi/tetrofosmin2	3.7–6.5	80	NA	-
	4–30 m (1 year)		6.5–12.6	80–87.92	0.053^b^	4.24–4.66
	31–90 m (5 years)		12.6–24.4	87.92–159.88	0.028^b^	2.46–4.48
	91–150 m (10 years)		24.4–42	159.88–255.92	0.018^b^	2.88–4.61
	151–210 m (15 years)		42–69.1	255.92–392	0.012^b^	3.07–4.70
	211–216 m (late adolescents)		> 69.1	≥ 392	0.009^b^	≥ 3.53
Renography standard	0–3 m (newborn)	^99m^Tc-labeled MAG3 (normal renal function, no accounting for emptying of bladder within 0.5–1 h post administration)	3.7–6.5	15–17.5	NA	-
	4–30 m (1 year)		6.5–12.6	17.5–25.9	0.022	0.38–0.57
	31–90 m (5 years)		12.6–24.4	25.9–37.8	0.012	0.31–0.45
	91–150 m (10 years)		24.4–42	37.8–52.5	0.012	0.45–0.63
	151–210 m (15 years)		42–69.1	52.5–68.7	0.009	0.47–0.62
	211–216 m (late adolescents)		> 69.1	≥ 68.7	0.007	≥ 0.48
Renal scintigraphy	0–3 m (newborn)	^99m^Tc-labeled DMSA	3.7–6.5	18.5	NA	-
	4–30 m (1 year)		6.5–12.6	18.5–21.3	0.037	0.68–0.79
	31–90 m (5 years)		12.6–24.4	21.3–38.8	0.021	0.45–0.82
	91–150 m (10 years)		24.4–42	38.8–62.1	0.015	0.58–0.93
	151–210 m (15 years)		42–69.1	62.1–95.2	0.011	0.68–1.05
	211–216 m (late adolescents)		> 69.1	≥ 95.2	0.0088	≥ 0.84

^a^Conversion factors from administered activity to effective dose [27]. ^b^Conversion factors are for sestamibi

^*99m*^*Tc* technetium-99 m, *DMSA* dimercaptosuccinic acid, *m* months, *MAA* macroaggregated albumin, *NA* not available, *sestamibi* methoxy isobutyl isonitrile, *TMAG3* mercaptoacetyl triglycine

**Fig. 2:**
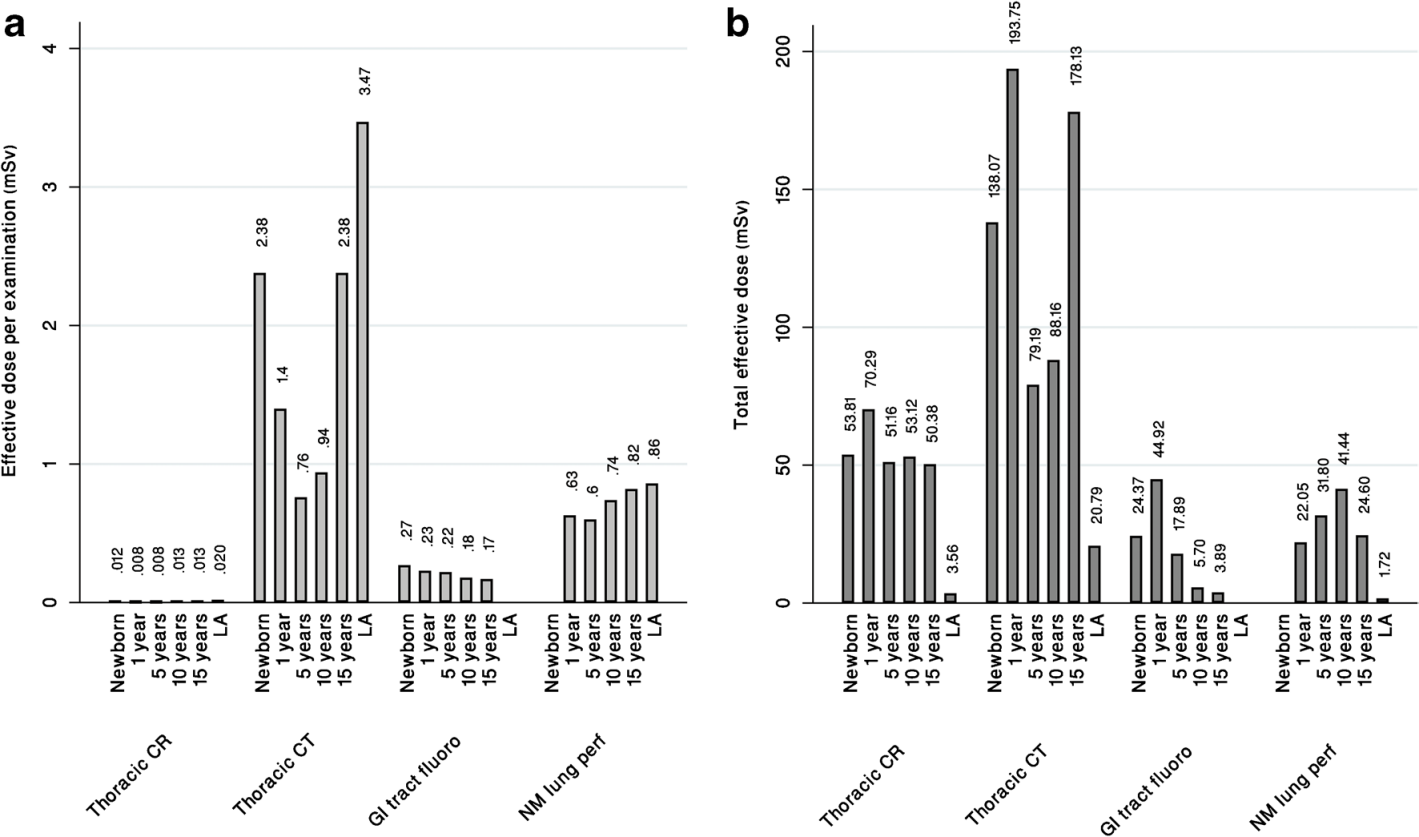
Comparison of effective dose per examination (**a**) and total effective dose (effective dose per examination multiplied by number of examinations) (**b**) over 20 years for six age categories and four modalities. *CR* conventional radiography, *CT* computed tomography, *GI* gastrointestinal, *LA* late adolescence, *NM* nuclear medicine, *perf* perfusion

Although conventional radiography of the thorax had a very low radiation dose per examination (about 0.008–0.02 mSv), it still contributed the second largest total effective dose after thoracic CT (about 0.75–3.5 mSv per examination) (Fig. 2): 50–70 mSv for conventional radiography vs. 79–194 mSv for CT within each age category. Thus, the total dose burden from conventional radiography ranged between 28–65% of the dose burden from thoracic CT in various age categories. For all ages combined, the dose contribution from thoracic conventional radiography was about 40% of that from thoracic CT.

Gastrointestinal tract fluoroscopy resulted in doses ranging from 0.17–0.27 mSv per examination. However, because the number of procedures was much lower than for conventional radiography, the total effective dose was approximately half for the two youngest categories and even lower for the older categories. Similarly, for NM lung perfusion, the effective dose per examination ranged from 0.6–0.86 mSv. However, the total effective dose from NM was lower than that for conventional radiography, for all age categories.

## Discussion

This study reports the frequency and dose contribution of conventional radiography, CT, diagnostic fluoroscopy, NM, and noncardiac intervention among Norwegian children and adolescents with CHD. Conventional radiography was the most frequent, accounting for 91% of all examinations. The dose contribution per examination from conventional radiography was low; however, the high number of examinations led to a considerable contribution to the total effective dose across all age groups. For thoracic examinations, it was found that thoracic CT had the highest dose per examination and total effective dose among all modalities. Because of frequent use, thoracic conventional radiography resulted in a total effective dose of about 40% of the contribution from thoracic CT. Since they were used less frequently, NM lung perfusion and gastrointestinal tract fluoroscopy contributed less to the total effective dose compared with thoracic conventional radiography. The thorax was the most frequent target for imaging, likely because of its association with cardiac disease. However, a high number of conventional radiography examinations were performed for scoliosis, and many CT examinations were performed on the head. NM examinations were performed less frequently with lung perfusion as the main examination.

As this was a group of patients with cardiac disorders, most examinations (about 85% of conventional radiography, 35% of CT scans, and around 58% of NM examinations) were performed on the thoracic region (Table 3). Several studies have highlighted the increasing trend and improved usefulness of CT in CHD diagnosis [9, 33–36]. In our study cohort, the use of CT was relatively limited, ranging from 5–7.5% across different age categories (Table 2). Among those patients who underwent CT, the average number of CT scans was approximately two (Table 3).

According to previous studies, CT is the imaging modality that contributes the most to radiation dose [13, 21]. Numerous studies have identified conventional radiography as having the highest number of examinations but making a relatively minor contribution to radiation dose [37, 38]. Vilar-Palop et al. reviewed the radiation dose from conventional radiography, CT, and diagnostic fluoroscopy for age groups similar to those in this current study [39]. They reported effective doses per thoracic conventional radiography ranging from 0.05–0.07 mSv and thoracic CT examination from 2.8–6.8 mSv. Additionally, they reported effective dose ranging from 0.7–5.8 mSv for gastrointestinal tract fluoroscopy examinations. In our study, the effective dose ranged from 0.008–0.02 mSv for thoracic conventional radiography, 0.76–3.47 mSv for thoracic CT, and 0.17–0.27 mSv for gastrointestinal tract fluoroscopy across different age categories (Fig. 2). Overall, the dose per examination in our study was generally lower than that in earlier studies [24, 27, 37, 39–45]. This could be attributed to a longer observation period (2000–2021), with several technological shifts that included improved dose control features, such as improved automatic exposure control technology, improved image processing and image reconstruction, and more sensitive detectors.

Previous studies have reported median effective doses for cardiac catheterization (both diagnostics and interventional), ranging from approximately 3–8 mSv [3, 4, 46–50]. In our study, the dose from thoracic conventional radiography was approximately 0.2% of the reported cardiac catheterization dose. Thoracic CT doses were approximately 25–45% of the reported effective doses from cardiac catheterization. Single NM procedures resulted in about 10–20% of the reported cardiac catheterization dose. It is worth noting that the dose from conventional radiography might seem negligible compared to cardiac catheterization procedures, but it may cause a substantial dose burden to patients if performed frequently.

A considerable number of conventional radiography examinations have been performed on the trunk region, mainly for scoliosis, in the 15 year age group. Several studies have documented an association between CHD and scoliosis [51–56]. Trunk examinations encompass the thorax, abdomen, and pelvis; thus, the average conversion factor for these three regions can be used to calculate the effective dose for trunk examinations. Despite a much lower number of conventional radiography examinations on the trunk compared to the thorax at 15 years, the total effective dose burden is almost equivalent. Therefore, trunk examinations should be considered an important contribution to the cumulative dose in this cohort.

The frequency of head CT examinations in our population is noteworthy. Studies have found that cerebral infarction and brain abscess are highly associated with cyanotic CHD, and these conditions are often detected using head/brain CT scans [57–60]. A case report by Zhang and Feng [61] showed an intracranial aneurysm resulting from complicated coarctation of the aorta, which was detected in a head CT scan. In addition, after a seizure episode following cardiac surgery, an initial head CT scan is often performed to ensure appropriate treatment [62].

Our cohort underwent several lung perfusion scintigraphy examinations. This finding is consistent with studies that have established a link between CHD and lung perfusion abnormalities [63–65]. Lung perfusion scintigraphy is the preferred method for the quantitative assessment of pulmonary perfusion in most patients with CHD [63–65].

We conducted this study as a part of HARMONIC delivery on the frequency and probability distribution of examinations performed on Norwegian patients with CHD. HARMONIC’s uncertainty estimate requires the probability of undergoing an ionizing radiation examination (provided in this work) as an input to obtain a more precise assessment of medical exposure beyond cardiac catheterization. In addition, the frequency distribution and dose magnitude will be of interest to clinicians, to inform them about the dose burden associated with different procedures. This provides knowledge of the potential risks associated with different imaging methods, aiding informed decisions on justification for each patient. This awareness might encourage proactive radiation optimization, such as using low-dose modalities, using non-ionizing radiation methods when suitable or reducing the number of acquisitions (such as performing one-view conventional radiography, if two views are not needed). Ultimately, this heightened awareness can enhance safety and personalization in medical practice, ensuring that imaging benefits outweigh the risks for pediatric patients.

There are limitations to this study. The most important is the possibility that some patients may have undergone examinations at hospitals other than Oslo University Hospital, which could have led to an underestimation of the radiation dose. Although Oslo University Hospital has the national responsibility for pediatric cardiology, examinations related to other conditions may have been performed at other hospitals. Furthermore, to estimate the effective dose from DAP and DLP, we used age-specific conversion factors based on the ICRP Publication 60 recommendations. These conversion factors are relatively crude and subject to considerable uncertainty, which could have affected the accuracy of our dose estimates, even though the effective doses provided in this study are only for comparison. However, the lack of conversion factors for some examinations makes it difficult to compare dose contributions from them. The effective dose for NM examinations uses population weight assumptions for the estimations of administrated activity, leading to higher error and inaccurate individual representation. In addition, the 2016 EANM dosage card may not reflect the actual dosage scheme of the historical study period.

## Conclusion

This study reports the frequency and radiation dose contribution of conventional radiography, CT, diagnostic fluoroscopy, NM, and noncardiac intervention among Norwegian patients with CHD and shows that CT was the biggest contributor to the radiation dose from imaging (excluding cardiac intervention). However, although the dose per conventional radiograph was low, the large number of examinations resulted in a total effective dose of approximately 40% of the CT. Therefore, it is important to consider the frequency of conventional radiography in addition to CT when considering patient exposure in this group. The findings of this study will help the HARMONIC project improve risk assessment by minimizing the uncertainty associated with cumulative dose calculations.

### Supplementary Information

Below is the link to the electronic supplementary material.

Supplementary file1 (PDF 144 KB) Table S1.1 The table provides an overview of the machines and manufacturers used at Oslo University Hospital for conventional radiography, computed tomography (CT) and angio/interventional procedures.

Supplementary file2 (PDF 275 KB) Table S2.1 The table presents the frequency of common conventional radiography procedures categorized by body region, along with corresponding dose values per examination for each age category for the Health Effects of Cardiac Fluoroscopy and Modern Radiotherapy in Pediatrics (HARMONIC) cohort of Norwegian patients with congenital heart disease. Table S2.2 The table presents the frequency of common computed tomography (CT) procedures categorized by body region, along with corresponding dose values per examination for each age category for the Health Effects of Cardiac Fluoroscopy and Modern Radiotherapy in Pediatrics (HARMONIC) cohort of Norwegian patients with congenital heart disease. Table S2.3 The table presents the frequency of common diagnostic fluoroscopy procedures categorized by body region, along with corresponding dose values per examination for each age category for the Health Effects of Cardiac Fluoroscopy and Modern Radiotherapy in Pediatrics (HARMONIC) cohort of Norwegian patients with congenital heart disease. Table S2.4 The table presents the frequency of noncardiac intervention procedures along with corresponding DAP and fluoroscopy time per examination for each age category for the Health Effects of Cardiac Fluoroscopy and Modern Radiotherapy in Pediatrics (HARMONIC) cohort of Norwegian patients with congenital heart disease

## Data Availability

The datasets generated during and/or analyzed during the current study are not publicly available because the data is primarily collected for project HARMONIC. According to Norwegian legislation, our approvals to use the data for the current study do not allow us to distribute or make the data directly available to other parties.
