# Supplementary material for: Use of ionizing radiation in a Norwegian cohort of children with congenital heart disease: imaging frequency and radiation dose for the Health Effects of Cardiac Fluoroscopy and Modern Radiotherapy in Pediatrics (HARMONIC) study
Source: Pediatr Radiol. 2023 Sep 29;53(12):2502–14. doi: 10.1007/s00247-023-05774-8 (PMC10635954; doi:10.1007/s00247-023-05774-8)
Supplement: Supplementary file 1 — Supplementary file1 (PDF 144 KB) Table S1.1 The table provides an overview of the machines and manufacturers used at Oslo University Hospital for conventional radiography, computed tomography (CT) and angio/interventional procedures. [file 247_2023_5774_MOESM1_ESM.pdf]

Article title

Use of ionizing radiation in a Norwegian cohort of children with congenital heart disease: Imaging frequency and radiation dose for the Health Effects of Cardiac Fluoroscopy and Modern Radiotherapy in Pediatrics (HARMONIC) study

Journal name

Pediatric Radiology

Author names

Susmita Afroz[1,2*], Bjørn H. Østerås[3], Utheya S. Thevathas[1,2], Gaute Dohlen[2], Caroline Stokke[3,4], Trude E. Robsahm[5], Hilde M. Olerud[1]

Affiliation

[1]Department of Optometry, radiography and lighting design, University of South-Eastern Norway, Drammen, Norway,

[2]Department of pediatric cardiology, Oslo University Hospital, Oslo, Norway

[3]Department of Physics and Computational Radiology, Oslo University Hospital, Oslo, Norway

[4]Dep. Of Physics, University of Oslo, Oslo, Norway

[5] Research department, Cancer Registry of Norway, Oslo, Norway

Email address of the corresponding author

Susmita.Afroz@usn.no

Table S1.1 Record of machines and manufacturers for each modality used in Oslo University Hospital (OUS)

Modality	Lab	Time Period	Machine	Manufacturer	Patients
Conventional radiography	Lab 1	Up to 2008	Philip Thoravision	Philips Healthcare, Amsterdam, Netherlands	Adult
		2008 – present	GE Definium 8000[a]	GE HealthCare, Chicago, IL	
	Lab 2	Up to 2016	Siemens Multix TOP (analogue)	Siemens Healthineers, Erlangen, Germany	Adult
		2016 – present	Adora (Canon detectors)	Canon Medical Systems, Tochigi, Japan	
	Lab 3	Up to 2018	Siemens TOP Flat Panel	Siemens Healthineers, Erlangen, Germany	Adult
		2018 – present	Siemens Ysio Max	Siemens Healthineers, Erlangen, Germany	
	Lab 4	Up to 2020	G.E. Prestige transillumination and exposure with CR / Film/foil	GE HealthCare, Chicago, IL	Adult
		2020 – present	Siemens Multitom RAX	Siemens Healthineers, Erlangen, Germany	
	Lab 6	Up to 2020	Siemens TOP Flat Panel	Siemens Healthineers, Erlangen, Germany	Adult
		2020 – present	Siemens Ysio Max	Siemens Healthineers, Erlangen, Germany	
	Lab 7	Up to 2019	Siemens TOP Flat Panel	Siemens Healthineers, Erlangen, Germany	Children
	Lab 8	Up to 2018	Philips diagnost 97 transillumination and exposure with CR	Philips Healthcare, Amsterdam, Netherlands	Children
		2018 – present	Siemens Artis Zee MP	Siemens Healthineers, Erlangen, Germany	Children
	Lab 9	Up to 2019	Philips MD 4 transillumination	Philips Healthcare, Amsterdam, Netherlands	Children
	Lab 11	2005 – 2018	Philips MD 4	Philips Healthcare, Amsterdam, Netherlands	
		2018 – present	Siemens Artis Zee MP	Siemens Healthineers, Erlangen, Germany	
	Mobile units	2000 – 2008	Siemens Mobilett and Siemens polymobile	Siemens Healthineers, Erlangen, Germany	
		2008 – 2017	Siemens Mobilett plus	Siemens Healthineers, Erlangen, Germany	
		2016 – present	Carestream DRX revolution	Carestream Health, Rochester, NY	
CT		2000 – 2004	GE HiSpeed single slice	GE HealthCare, Chicago, IL	
		2004 – 2006	GE LightSpeed Pro 16 slice	GE HealthCare, Chicago, IL	
		2006 – 2011	GE VCT 64 slice	GE HealthCare, Chicago, IL	

	2011 – 2017	Toshiba Aquilion One 320 Slice	Toshiba, Tokyo, Japan	
	2017 – present	Siemens Somatom Force Dual Source 2x 198 slice	Siemens Healthineers, Erlangen, Germany	
Angio/ Intervention	2000 – 2011	Siemens Bicor with image intensifier	Siemens Healthineers, Erlangen, Germany	Children
	2011 – present	Siemens Artis Zee Biplane system with a digital flat panel detector.		Children

[a]For thorax imaging

CR computed radiography, *CT* computed tomography
