# Supplementary material for: Use of ionizing radiation in a Norwegian cohort of children with congenital heart disease: imaging frequency and radiation dose for the Health Effects of Cardiac Fluoroscopy and Modern Radiotherapy in Pediatrics (HARMONIC) study
Source: Pediatr Radiol. 2023 Sep 29;53(12):2502–14. doi: 10.1007/s00247-023-05774-8 (PMC10635954; doi:10.1007/s00247-023-05774-8)
Supplement: Supplementary file 2 — Supplementary file2 (PDF 275 KB) Table S2.1 The table presents the frequency of common conventional radiography procedures categorized by body region, along with corresponding dose values per examination for each age category for the Health Effects of Cardiac Fluoroscopy and Modern Radiotherapy in Pediatrics (HARMONIC) cohort of Norwegian patients with congenital heart disease. Table S2.2 The table presents the frequency of common computed tomography (CT) procedures categorized by body region, along with corresponding dose values per examination for each age category for the Health Effects of Cardiac Fluoroscopy and Modern Radiotherapy in Pediatrics (HARMONIC) cohort of Norwegian patients with congenital heart disease. Table S2.3 The table presents the frequency of common diagnostic fluoroscopy procedures categorized by body region, along with corresponding dose values per examination for each age category for the Health Effects of Cardiac Fluoroscopy and Modern Radiotherapy in Pediatrics (HARMONIC) cohort of Norwegian patients with congenital heart disease. Table S2.4 The table presents the frequency of noncardiac intervention procedures along with corresponding DAP and fluoroscopy time per examination for each age category for the Health Effects of Cardiac Fluoroscopy and Modern Radiotherapy in Pediatrics (HARMONIC) cohort of Norwegian patients with congenital heart disease [file 247_2023_5774_MOESM2_ESM.pdf]

Article title

Use of ionizing radiation in a Norwegian cohort of children with congenital heart disease: Imaging frequency and radiation dose for the Health Effects of Cardiac Fluoroscopy and Modern Radiotherapy in Pediatrics (HARMONIC) study

Journal name

Pediatric Radiology

Author names

Susmita Afroz[1,2*], Bjørn H. Østerås[3], Utheya S. Thevathas[1,2], Gaute Dohlen[2], Caroline Stokke[3,4], Trude E. Robsahm[5], Hilde M. Olerud[1]

Affiliation

[1]Department of Optometry, radiography and lighting design, University of South-Eastern Norway, Drammen, Norway,

[2]Department of pediatric cardiology, Oslo University Hospital, Oslo, Norway

[3]Department of Physics and Computational Radiology, Oslo University Hospital, Oslo, Norway

[4]Dep. Of Physics, University of Oslo, Oslo, Norway

[5] Research department, Cancer Registry of Norway, Oslo, Norway

Email address of the corresponding author

Susmita.Afroz@usn.no

Table S2.1 Record of dose area product (DAP) and effective dose values in common conventional radiography examinations stratified by body region for the Health Effects of Cardiac Fluoroscopy and Modern Radiotherapy in Pediatrics (HARMONIC) cohort of Norwegian patients with congenital heart disease

Body region	Age category	Number of examinations	Number of examinations with DAP	Median DAP (Gycm^2)	DAP IQR (Gycm^2)	Conversion factor: effective dose/DAP (mSv/Gycm^2)[a]	Median (IQR) effective dose (mSv)
Neck (AP/Lat)	0–3 m (newborn)	3	1	0.04	-	NA	-
	4–30 m (1 year)	16	8	0.02	0.01–0.02	NA	-
	31–90 m (5 years)	29	9	0.03	0.03–0.11	NA	-
	91–150 m (10 years)	20	10	0.12	0.02–0.21	NA	-
	151–210 m (15 years)	11	7	0.08	0.04–0.10	NA	-
	211–216 m (late adolescents)	0	0	-	-	NA	-
Thorax (AP/PA)	0–3 m (newborn)	4,615	428	0.01	0.004–0.01	2.20	0.012 (0.01–0.02)
	4–30 m (1 year)	9,322	2,376	0.01	0.01–0.02	0.58	0.008 (0.005–0.01)
	31–90 m (5 years)	6,567	2,780	0.02	0.01–0.04	0.41	0.008 (0.004–0.02)
	91–150 m (10 years)	4,216	1,910	0.05	0.03–0.07	0.28	0.013 (0.01–0.02)
	151–210 m (15 years)	3,840	1,323	0.08	0.06–0.12	0.16	0.013 (0.01–0.02)
	211–216 m (late adolescents)	177	19	0.13	0.1–0.17	0.15	0.020 (0.02–0.03)
Abdomen (AP)	0–3 m (newborn)	250	22	0.01	0.01–0.01	2.00	0.016 (0.01–0.03)
	4–30 m (1 year)	90	28	0.02	0.01–0.04	0.91	0.019 (0.012–0.04)
	31–90 m (5 years)	56	16	0.04	0.03–0.09	0.64	0.026 (0.02–0.06)
	91–150 m (10 years)	55	25	0.05	0.04–0.19	0.40	0.021 (0.02–0.08)
	151–210 m (15 years)	43	23	0.80	0.26–1.25	0.28	0.224 (0.07–0.35)
	211–216 m (late adolescents)	0	0	-	-	0.20	-
Pelvis (AP)	0–3 m (newborn)	10	4	0.01	0.004–0.01	2.20	0.013 (0.01–0.02)
	4–30 m (1 year)	27	9	0.01	0.01–0.02	1.10	0.012 (0.01–0.02)
	31–90 m (5 years)	30	13	0.03	0.02–0.05	0.60	0.020 (0.01–0.03)
	91–150 m (10 years)	43	18	0.11	0.04–0.33	0.28	0.029 (0.01–0.09)
	151–210 m (15 years)	62	26	0.19	0.11–0.48	0.21	0.039 (0.02–0.10)
	211–216 m (late adolescents)	3	3	0.24	0.22–0.65	0.22	0.052 (0.05–0.14)
Limbs	0–3 m (newborn)	26	2	0.02	0.02–0.02	NA	-
	4–30 m (1 year)	67	18	0.01	0.003–0.01	NA	-
	31–90 m (5 years)	127	46	0.01	0.002–0.02	NA	-
	91–150 m (10 years)	203	94	0.02	0.004–0.04	NA	-
	151–210 m (15 years)	214	113	0.02	0.01–0.07	NA	-
	211–216 m (late adolescents)	17	11	0.05	0.01–0.12	NA	-
Trunk (PA)	0–3 m (newborn)	3	1	0.04	-	NA	-
	4–30 m (1 year)	10	5	0.01	0.004–0.02	NA	-
	31–90 m (5 years)	66	29	0.03	0.01–0.06	NA	-
	91–150 m (10 years)	143	83	0.14	0.07–0.26	NA	-
	151–210 m (15 years)	306	172	0.50	0.28–0.75	NA	-
	211–216 m (late adolescents)	15	8	0.40	0.21–0.54	NA	-

[a]Conversion factors from DAP to effective dose [27]

AP anterior to posterior *DAP d*ose area product, *IQR* interquartile range, *Lat* Lateral, *m* months, *NA* not available, *PA* posterior to anterior

Table S2.2 Record of dose length product (DLP) and effective dose values in common computed tomography (CT) examinations stratified by body region for the Health Effects of Cardiac Fluoroscopy and Modern Radiotherapy in Pediatrics (HARMONIC) cohort of Norwegian patients with congenital heart disease

Body region	Age category	Number of examinations	Number of examination with DAP	Median DLP (mGycm)	DLP IQR (mGycm)	Conversion factor: effective dose/ DLP (mSv/mGycm)[a]	Median (IQR) effective dose (mSv)
CT head	0–3 m (newborn)	23	5	236.00	61–287	0.011	2.59 (0.67–3.16)
	4–30 m (1 year)	64	12	384.45	339–497.71	0.007	2.58 (2.27–3.33)
	31–90 m (5 years)	69	33	520.94	157.68–580.54	0.004	2.08 (0.63–2.32)
	91–150 m (10 years)	76	47	557.26	123.89–715.29	0.003	1.78 (0.39–2.29)
	151–210 m (15 years)	72	40	588.92	125.32–823.19	0.002	1.24 (0.26–1.73)
	211–216 m (late adolescents)	3	1	179.90	-	0.002	0.38(NA)
CT thorax	0–3 m (newborn)	58	27	61.04	43.1–65.65	0.039	2.38 (1.68–2.56)
	4–30 m (1 year)	138	51	54.00	23–88.65	0.026	1.40 (0.59–2.30)
	31–90 m (5 years)	104	48	42.30	25.81–69.73	0.018	0.76 (0.46–1.26)
	91–150 m (10 years)	94	44	72.14	36.355–126.65	0.013	0.94 (0.47–1.65)
	151–210 m (15 years)	75	41	169.65	58–248.15	0.014	2.38 (0.81–3.47)
	211–216 m (late adolescents)	6	2	247.5	154–341	0.014	3.47 (2.16–4.77)
CT abdomen	0–3 m (newborn)	6	1	34.31	-	0.049	1.68 (NA)
	4–30 m (1 year)	9	1	157.00	-	0.030	4.71 (NA)
	31–90 m (5 years)	11	7	91.21	51–110.16	0.020	1.82 (1.02–2.20)
	91–150 m (10 years)	21	8	149.73	127.09–184	0.015	2.25 (1.91–2.76)
	151–210 m (15 years)	13	7	470.00	206.33–1075	0.015	7.05 (3.09–16.13)
	211–216 m (late adolescents)	0	0	-	-	0.015	-

[a]Conversion factors from DLP to effective dose [28]

CT computed tomography, *DLP d*ose length product, *IQR* interquartile range, *m* months, *NA* not available

Table S2.3 Record of dose area product (DAP) and effective dose values in common diagnostic fluoroscopy examinations stratified by body region for the Health Effects of Cardiac Fluoroscopy and Modern Radiotherapy in Pediatrics (HARMONIC) cohort of Norwegian patients with congenital heart disease

Body region	Age category	Number of examinations	Number of examinations with DAP	Median DAP (Gycm^2)	DAP IQR (Gycm^2)	Median fluoro time (min)	Conversion factor: effective dose/DAP (mSv/Gycm^2)[a]	Median (IQR) effective dose (mSv)
Gastrointestinal tract (neck + chest +abdomen)	0–3 m (newborn)	91	74	0.15	0.07–0.27	3.3	1.79[b]	0.27 (0.12–0.48)
	4–30 m (1 year)	197	121	0.30	0.15–0.50	2.8	0.76[b]	0.23 (0.11–0.38)
	31–90 m (5 years)	83	63	0.44	0.19–0.78	2.4	0.49[b]	0.22 (0.09–0.38)
	91–150 m (10 years)	32	25	0.54	0.18–0.99	2.8	0.33[b]	0.18 (0.06–0.33)
	151–210 m (15 years)	23	15	0.77	0.28–1.24	2.1	0.22[b]	0.17 (0.06–0.27)
	211–216 m (late adolescents)	0	0	-	-	-	0.2[b]	-
Uro-genital tract	0–3 m (newborn)	13	9	0.14	0.07–0.20	2.0	1.28	0.18 (0.09–0.26)
	4–30 m (1 year)	30	15	0.34	0.17–0.68	2.1	0.64	0.22 (0.11–0.44)
	31–90 m (5 years)	36	15	0.22	0.19–0.48	1.9	0.46	0.10 (0.09–0.22)
	91–150 m (10 years)	51	18	0.48	0.34–0.84	1.6	0.32	0.15 (0.11–0.27)
	151–210 m (15 years)	11	5	0.74	0.69–2.16	3.3	0.20	0.15 (0.14–0.43)
	211–216 m (late adolescents)	0	0	-	-	-	0.20	-
Angiography (excluding heart angio)	0–3 m (newborn)	9	9	1.37	1.13–6.02	19.7	NA	-
	4–30 m (1 year)	34	21	2.46	0.67–3.80	16.2	NA	-
	31–90 m (5 years)	18	12	8.23	3.66–22.02	15.0	NA	-
	91–150 m (10 years)	12	4	9.86	2.92–26.22	21.3	NA	-
	151–210 m (15 years)	22	17	16.73	7.20–38.48	15.2	NA	-
	211–216 m (late adolescents)	0	0	-	-	-	NA	-

[a]Conversion factors from DAP to effective dose [27]. [b]Conversion factor for gastrointestinal track is the average of factors for stomach & duodenum and Oesophagus [27]

*DAP d*ose area product, *IQR* interquartile range, *m* months, *NA* not available

Table S2.4 Record of dose area product (DAP) values in noncardiac interventional procedures for the Health Effects of Cardiac Fluoroscopy and Modern Radiotherapy in Pediatrics (HARMONIC) cohort of Norwegian patients with congenital heart disease

Exam	Age category	Number of examinations	Number of examinations with DAP	Median DAP ($Gycm^2$)	DAP IQR ($Gycm^2$)	Median fluoro time (min)	Conversion factor: effective dose/DAP (mSv/ $Gycm^2$)
Noncardiac Intervention	0–3 m (newborn)	16	7	0.21	0.08–4.2	1.4	NA
	4–30 m (1 year)	29	18	0.72	0.21–3.19	2.95	NA
	31–90 m (5 years)	22	12	0.63	0.41–11.16	2.9	NA
	91–150 m (10 years)	7	5	1.70	0.09–11.55	18.2	NA
	151–210 m (15 years)	24	16	13.25	2.19–27.85	13	NA
	211–216 m (late adolescents)	1	1	40.19	-	22.7	NA

*DAP d*ose area product, *IQR* interquartile range, *m* months, *NA* not available
